# Acute bilateral cerebral infarction in the presence of neuromyelitis optica spectrum disorder: A case report

**DOI:** 10.1097/MD.0000000000022616

**Published:** 2020-10-02

**Authors:** Zi-Yi Wang, Meng Wang, Jiao-Jiao Guo, Yan-Lu Gao, Xue-Fan Yu

**Affiliations:** Department of Neurology, The First Hospital of Jilin University, Hongqi Street, Changchun, Jilin, China.

**Keywords:** case report, cerebral infarction, magnetic resonance imaging, methylprednisolone, neuromyelitis optica spectrum disorders

## Abstract

**Rationale:**

Neuromyelitis optica spectrum disorders (NMOSDs) are inflammatory demyelinating disorders of the central nervous system; they are characterized by severe optic neuritis and transverse myelitis. Intravenous methylprednisolone pulse (IVMP) therapy is an effective treatment that is administered to patients in the acute phase of NMOSD; this therapy has achieved remarkable results in clinical practice. However, there are no reports on NMOSD patients who have experienced an acute bilateral cerebral infarction while undergoing IVMP treatment.

**Patient concerns:**

We report on a 62-yr-old woman who was undergoing IVMP therapy for the primary diagnosis of NMOSD. Unexpectedly, the patient's existing limb weakness worsened, and she developed motor aphasia on the second day of IVMP treatment. Additionally, brain magnetic resonance imaging revealed acute bilateral cerebral infarction.

**Diagnosis:**

The patient's clinical manifestations, medical imaging results, and laboratory test results were taken into consideration; the final diagnosis was acute bilateral cerebral infarction in the presence of NMOSD.

**Interventions:**

Subsequent to the onset of acute cerebral infarction, the patient was immediately treated with oral aspirin, atorvastatin, and intravenous butylphthalide. The hormone dose was adjusted to an oral 60-mg/d dose for maintenance; this was followed by immunoadsorption plasmapheresis for 3 days, and double-filtration plasmapheresis for 2 days.

**Outcomes:**

Following treatment onset, the patient's ocular symptoms significantly improved, and her limb muscle strength gradually recovered. Two months after discharge, the patient's husband reported that she was able to walk with the help of others and take care of herself, and that there was no recurrence.

**Lessons:**

Medical professionals must be aware of the possibility of NMOSD patients with cerebrovascular risk factors suffering an acute cerebral infarction while undergoing high-dose IVMP therapy, as this therapy can exacerbate existing problems.

## 1 Introduction

Neuromyelitis optica spectrum disorders (NMOSDs) are immune-mediated inflammatory disorders of the central nervous system that are characterized by the targeting of optic nerves and the spinal cord, and severe demyelination and axonal damage, which are primarily mediated by humoral immunity; particularly, these problems are closely related to serum aquaporin-4 (AQP4) antibodies.^[1]^ IVMP therapy is generally a preferred treatment for acute NMOSD attacks, as it has previously been demonstrated to effectively reduce inflammatory responses.^[2]^ However, IVMP therapy has recently been reported to be associated with acute stress events.^[3]^ Particularly, high-dose IVMP treatments have been associated with a risk of liver cell damage; moreover, cases of liver failure associated with IVMP therapy have been reported to result in patient fatality.^[4]^ There has also been a case of a patient experiencing acute myocardial infarction during high-dose methylprednisolone therapy for Graves ophthalmopathy.^[5]^ The probability of NMOSD co-occurring with acute cerebral infarction is very low, and there are no existing reports on such a case. Thus, we report on a rare case of NMOSD comorbid with acute bilateral cerebral infarction resulting from IVMP treatment.

## 2 Case presentation

A 62-yr-old Chinese woman was admitted to our hospital with complaints of chest tightness, constipation, urinary incontinence, and lower-extremity pain and numbness that had caused her to reduce her walking speed for 5 days. In addition to the lower-extremity pain, she reported weakness in her upper and lower limbs. The symptoms rapidly progressed, and were accompanied by blurred vision in her left eye within 1 day. We reviewed her medical history and learned that she was diagnosed with hypertension and hyperlipidemia approximately 10 years before the current admittance, and that she had been adhering to an oral treatment regimen that included nifedipine and atorvastatin calcium. She had no family history of neurological diseases, and denied any history of alcohol or substance abuse, smoking, or drinking. Additionally, she was fully alert during her neurological examination, showing no signs of disorientation. Cranial nerve evaluation revealed no abnormalities except for blurred vision in the left eye. According to the muscle strength grading scale, the muscle strength of all limbs was Grade 3 (maximum score: 5) under the conditions of normal muscular tension. The tendon reflexes were brisk in both lower limbs, but normal in the upper extremities. Acupuncture hypoesthesia was observed below the level of T2, and the bilateral Babinski and Chaddock signs were positive. The patient underwent further physical examination, but the results were unremarkable.

**Figure 1:**
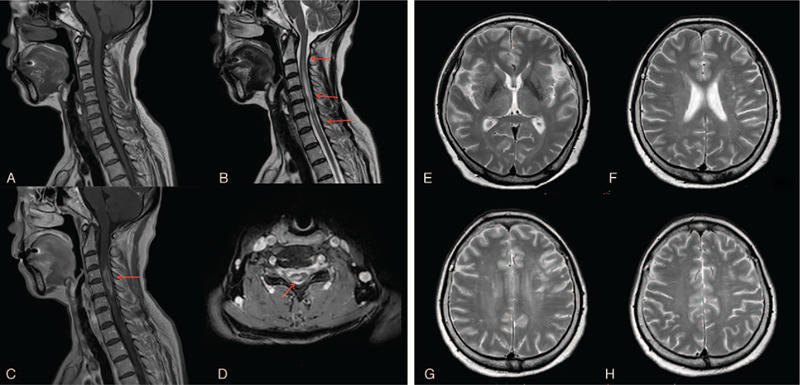
Sagittal T1-weighted (A) T2-weighted (B) images of the spine revealed a longitudinally extensive lesion extending from C2 to C7 vertebrae (red arrows, A–B); an enhanced irregular sign can be observed at the C3 to C5 level (red arrow, D). Axial cervical magnetic resonance imaging (MRI) images reveal a mass lesion mainly located in the center of the cord (red arrow, C). Initial brain MRI results confirmed the absence of any abnormality prior to intravenous methylprednisolone treatment (E–H).

**Figure 2:**
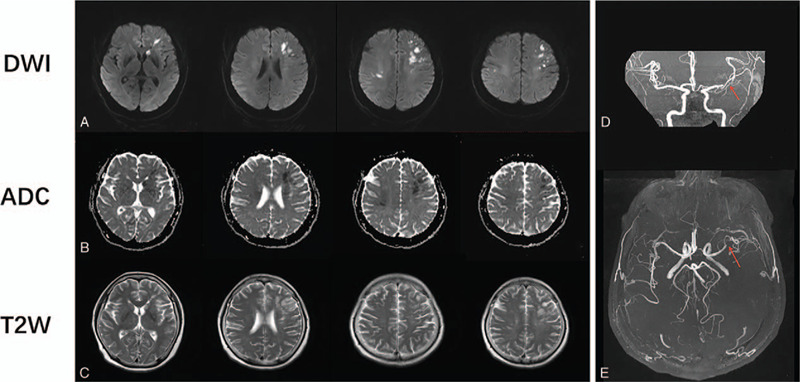
Repeat brain MRI (2 days after intravenous methylprednisolone treatment) results confirm an acute infarction in both frontal lobe hemispheres, the left basal ganglia, and the semioval center (bilateral). Diffusion weighted imaging results confirmed the presence of high-signal lesions (A); the T2-weighted results also revealed a slightly hyperintense signal (C). The corresponding lesions present as a hypointense signal on the apparent diffusion coefficient sequence. Magnetic resonance angiography revealed the existence of localized stenosis in the M1 segment of the left middle cerebral artery, as well as multiple intracranial arteriosclerosis (red arrow, D–E).

Routine blood tests confirmed a high homocysteine level of 46 μm (normal: 0–20 μm), and low-density lipoprotein cholesterol of 3.93 mmol/L (normal: 0–3.28 mmol/L). A cerebrospinal fluid (CSF) examination confirmed that the patient had an intracranial pressure of 160 mm H_2_O, a protein mass concentration of 1.69 g/L (normal: 0.15–0.45 g/L), an immunoglobulin G (IgG) mass concentration of 134 mg/L (normal: 0–34 mg/L), a white blood cell count of 18 × 10^6^/L (normal: 0–8 × 10^6^/L), and normal glucose and iron levels. This study was approved by the ethics committee of the First Hospital of Jilin University in Changchun, China. Informed, written consent was obtained from the patient for publication of this case report. The cytometric beads array method was used to analyze the serum and CSF; the results revealed the presence of AQP4 antibodies, and the absence of oligoclonal bands and myelin oligodendrocyte glycoprotein antibodies in the serum and CSF. Serological assays for autoimmune diseases and connective tissue diseases that affect the thyroid and/or antinuclear antibodies yielded negative results. The results of visual evoked potential (VEP) analysis confirmed relatively low P100 wave amplitudes with prolonged latency in both eyes. Cervical spine magnetic resonance imaging (MRI) revealed the existence of a longitudinally extensive hypointensive lesion extending from the C2 to C7 vertebrae (Fig. 1). Post-contrast-enhanced MRI results revealed clear and irregular enhancement (Fig. 1). However, no abnormal signal was found in the brain MRI results (Fig. 1). Thus, in consideration of the clinical features, laboratory test results, and VEP and MRI findings, a diagnosis of NMOSD was confirmed. The patient began a high-dose IVMP treatment (1000 mg/d) immediately following diagnosis. The patient was expected to continue this treatment for a period of 3 d. However, her bilateral limb muscle strength suddenly decreased, and she developed motor aphasia on the second day of IVMP treatment. Specifically, the muscle strength of her upper limbs decreased to Grade 2; her National Institutes of Health Stroke Scale (NIHSS) score was 18. Repeat brain MRI revealed that the patient had suffered an acute infarction in both frontal lobe hemispheres, the left basal ganglia, and bilateral semioval center (Fig. 2). Magnetic resonance angiography (MRA) confirmed localized stenosis in the M1 segment of the left middle cerebral artery, as well as multiple intracranial arteriosclerosis (Fig. 2). After reviewing these results, we immediately discontinued IVMP therapy, and initiated a treatment consisting of oral aspirin, atorvastatin calcium, and intravenous butylphthalide. Then, we initiated a 3-days immunoadsorption plasmapheresis treatment, and 2-days double-filtration plasmapheresis treatment. We also began administering oral prednisolone at 60 mg/d as a maintenance treatment. At the time of discharge, the patient's speech disorder and urinary dysfunction had slightly improved; she could also clearly see close objects, but did not fully recover her muscle strength, as her NIHSS score was 9. Two months after discharge, the patient's husband stated she was able to walk with the help of others and take care of herself, and that there was no recurrence.

## 3 Discussion

In the present case, the patient was initially diagnosed with NMOSD based on standard diagnostic criteria; standard IVMP therapy was subsequently implemented. However, during the IVMP treatment, the patient's limb weakness suddenly worsened, and she developed motor aphasia, which aggravated her condition. It is worth noting that the new symptom of motor aphasia facilitated the diagnosis of acute cerebral infarction. Diffusion weighted imaging was applied to detect brain lesions within 6 h of her acute infarction, and to distinguish between new and old infarcts. The results revealed that her bilateral cerebral infarction exacerbated her NMOSD symptoms, consequently further reducing her bilateral limb muscle strength. Because her clinical manifestations differed from those associated with limb hemiplegia, which is commonly associated with unilateral cerebral infarction, her clinical diagnosis was difficult. This patient had a history of severe hypertension and hyperlipidemia for about 10 years, which contributed greatly to the cerebral arteriosclerosis and vascular damage. MRA results revealed a localized narrowing of the left middle cerebral artery and bilateral extensive cerebral atherosclerosis that was consistent with the patient's ischemic location. We believe that the localized narrowing of the left middle cerebral artery and extensive cerebral arteriosclerosis were the primary causes of this cerebral infarction, and that the IVMP therapy, which was prescribed to treat her NMOSD, acted as a trigger. To the best of our knowledge, this is the first report on the co-occurrence of NMOSD and acute bilateral cerebral infarction where antiplatelet aggregation therapy and plasmapheresis proved effective.

The binding of NMO-IgG to astrocyte AQP4 is directly involved in the pathogenesis of most patients with NMOSD; it leads to astrocyte loss, secondary inflammatory infiltration, demyelination, and neuronal death.^[6]^ In a rat model, AQP4 antibodies have been demonstrated to worsen edema in the event of an ischemic stroke.^[7]^ Furthermore, AQP4 antibodies may also damage the structural integrity of astrocytes by way of some immunoregulatory pathway, such as the activation of the classical complement cascade, or granulocyte recruitment, which consequently increases blood brain barrier (BBB) permeability.^[8]^ It should also be noted that AQP4-dependent water flow through the cell membrane is an important mechanism for angioedema alleviation. Specifically, AQP4 antibodies can play a role in inhibiting AQP4 expression, which alleviates edema.^[9]^ Once a cerebral infarction occurs, AQP4 antibodies can also down-regulate the expression of the glutamate transporter EAAT2, which causes glutamate circulation disorders and results in a more severe infarction.^[7]^

IVMP therapy has been widely implemented in the acute phase of NMOSD for many years owing to the fact that corticosteroids reduce the inflammatory response, and that the standard dose is 1,000 mg for 3 to 5 consecutive days.^[10]^ However, adverse events, such as infection, abdominal pain, hypocalcemia, hypofibrinogen, weight gain, and hyperglycemia have been reported as side effects of IVMP therapy.^[11]^ Corticosteroids play an important role in enhancing vascular tone, primarily through the strengthening of the effects of vasoconstrictor hormones such as angiotensin and endoderin, as well as their direct effects on vascular smooth muscle cells (VSMCs); these conditions lead to disease deterioration. Glucocorticoids can also promote the formation of angiotensin II (Ang II), and up-regulate Ang II binding receptors in blood vessels, which further potentiate Ang II vasoconstrictor action.^[12]^ Furthermore, glucocorticoids can bind to glucocorticoid receptors in VSMCs, thereby increasing the sensitivity of blood vessels to adrenergic agonists, and promoting further vasoconstriction.^[13]^ Alternatively, they can also increase the proliferation of VSMCs, which promotes nutritive activity and leads to enhanced vasoconstriction.^[14]^ A study on the adverse prognostic factors in patients with reversible cerebral vasoconstriction syndrome revealed that methylprednisolone can worsen the patient's condition within 2 to 6 days after treatment onset.^[15]^ Once a cerebral infarction occurs, AQP4 antibodies may worsen cerebral edema, and even initiate a series of stress responses that damage the BBB. Moreover, cerebral hemorrhaging may be a consequence of BBB disruption that occurs as a patient's blood pressure increases in response to high-dose hormones. It should be noted that there have been reports of cerebral hemorrhaging in NMOSD patients who were undergoing IVMP therapy.^[16]^ All of the above-mentioned findings provide the theoretical basis for the following hypotheses:

(1) along with the invasion of the vessels by the AQP4 antibodies, IVMP treatments can constrict the cerebral blood vessels by series of pathways;
(2) in this patient, who had hyperlipidemia, atherosclerosis, and artery stenosis of the brain, the constriction of the blood vessels and damage to the vascular endothelial cells in the brain caused by IVMP therapy (particularly early after initiation) can induce acute cerebral infarction.

We have reported on a rare case of an individual with NMOSD who suffered a bilateral infarction during IVMP therapy. We have emphasized that medical professionals should take into consideration the fact that an acute cerebrovascular event is possible if the symptoms of NMOSD patients worsen, or if they suddenly develop new symptoms. In such cases, intensive immunotherapy and antiplatelet aggregation therapy, which enhances cerebral circulation, could be effective. Furthermore, we also recommend against high-dose IVMP therapy since it is not considered completely safe for the treatment of patients with NMOSD who have identified risk factors for cerebrovascular disease, such as cerebrovascular stenosis, severe cerebral arteriosclerosis, and history of stroke.

## Acknowledgments

We are grateful to Editage for English language editing.

## Author contributions

Study conceptualization and design, drafting of the manuscript: ZYW and MW

Acquisition of data: JJG; MW and YLG

Decision to publish: JJG and MW

Critical revision of manuscript for intellectual content: XFY and ZYW

**Conceptualization:** Zi-Yi Wang.

**Data curation:** Jiao-Jiao Guo, Yan-Lu Gao.

**Funding acquisition:** Xuefan Yu.

**Investigation:** Jiao-Jiao Guo, Yan-Lu Gao.

**Resources:** Zi-Yi Wang.

**Software:** Jiao-Jiao Guo, Yan-Lu Gao.

**Supervision:** Xuefan Yu.

**Writing – original draft:** Zi-Yi Wang, Meng Wang.

**Writing – review and editing:** Xuefan Yu.
